# Investigation of three-rooted deciduous mandibular second molars in a Chinese population using cone-beam computed tomography

**DOI:** 10.1186/s12903-022-02378-w

**Published:** 2022-08-08

**Authors:** Chengfeng Jiang, Fan Pei, Yihan Wu, Yifen Shen, Ying Tang, Xingmei Feng, Yongchun Gu

**Affiliations:** 1 Department of Dentistry and Central Laboratory, Ninth People’s Hospital of Suzhou, Soochow University, Ludang Road 2666#, Wujiang Dist., Suzhou, 215200 China; 2 Department of Stomatology, Affiliated Hospital of Nantong University, Nantong, 226006 China

**Keywords:** Three-rooted deciduous mandibular second molar, Three-rooted permanent mandibular first molar, Disto-lingual root, Root curvature, Cone-beam computed tomography

## Abstract

**Background:**

To investigate the anatomic features of three-rooted deciduous mandibular second molars (DMSMs) in Chinese children by using cone-beam computed tomography (CBCT).

**Methods:**

A total of 247 CBCT scans of Chinese children were selected and retrospectively analyzed. The occurrence, gender and side predilection of three-rooted DMSMs were examined. The pattern of concurrence of bilateral three-rooted DMSMs, and concurrence of three-rooted DMSM and three-rooted permanent mandibular first molar (PMFM) was analyzed by the concurrence rate and Spearman’s rank correlation test. The geometric parameters of the disto-buccal (DB) and disto-lingual (DL) roots, including the vertical root length, level and angle of distal root furcation, angle of root curvature (by Schneider technique) and the spreading angle, were measured and compared to the three-rooted PMFMs (n = 42) from 100 randomly selected adult subjects.

**Results:**

The occurrence of three-rooted DMSMs was 24.0% (54/225) calculated by individual, and 18.6% (88/472) by tooth. A significant right-side predilection was detected (23.0% vs 14.2%, *p* < 0.05), while gender predilection was not detected (*p* > 0.05). The bilateral concurrence rate was 49.0%, and Spearman’s correlation test indicated a significant relationship between the antimetric teeth (*rho* = 0.609, *p* < 0.01); whereas a weak but significant co-relationship was detected between the three-rooted DMSM and three-rooted PMFM (right side: concurrence rate = 31.6%, *rho* = 0.325, *p* < 0.01; left side: concurrence rate = 23.0%, *rho* = 0.260, *p* < 0.01). The length of DL roots in the DMSMs was 7.4 ± 1.5 mm, and the curvature angle was 16.4 ± 11.3 degrees, which was significantly (both *p* < 0.01) lower than that of the three-rooted PMFMs (root length = 11.0 ± 1.3 mm; degrees of curvature = 34.2 ± 16.1 degrees), whereas the spreading angle of the DL root in DMSMs (34.6 ± 8.4 degrees) was significantly (*p* < 0.01) greater than in the PMFMs (26.8 ± 6.5 degrees).

**Conclusions:**

Three-rooted DMSMs have a high occurrence rate in the Chinese children with a right-side predilection, and they have a weak but statistically significant correlation with three-rooted PMFMs. The DL roots of DMSMs are shorter, less curved, and spreading more widely as compared with those in the three-rooted PMFMs.

## Background

The deciduous mandibular second molar (DMSM) and permanent mandibular first molar (PMFM) are similar in morphology, and they typically have two roots (one mesial and one distal); but in some cases, an extra root may occur on the disto-lingual (DL) side, which was also called radix entomolaris in the literature [1, 2]. In dental anthropology, this root variation is regarded as an important ethnic characteristic for Mongoloid populations, with a high prevalence rate ranging from 5 to 40%; whereas in black or white populations, the frequency is mostly less than 5% [2–7].

In dentistry, the presence of an DL root may pose a challenge for endodontic treatment [1, 2]. Because it is often superimposed by the disto-buccal (DB) root in the periapical radiographs, ignorance of its presence by the clinicians may lead to treatment failure [8]. Moreover, the DL canal is frequently tiny in size and severely curved from the proximal view, which is more prone to procedural errors or instrument separation during root canal instrumentation [9, 10]. Therefore, the DL root has attracted a lot of research attention. However, to date, few scholars have fully investigated the anatomic features of the three-rooted DMSMs [11]. The reasons may be as follows: (1) For ex vivo studies which usually based on transparent technique or micro-computed tomography, collection of a large sample size of non-carious DMSMs without root resorption is difficult; moreover, the greater root divergence of deciduous molars [12] is prone to root fracture during tooth extraction; (2) most previous in vivo studies [11, 13] have relied on conventional periapical radiography or panoramic radiography, which are two-dimensional, and can hardly display the three-dimensional (3D) shape of the DL root; (3) it is often difficult to obtain high-quality diagnostic radiograph of deciduous teeth due to young age and often poor cooperation.

In recent years, dental cone-beam computed tomography (CBCT) has been widely used to evaluate the patients’ root and canal anatomy due to its high resolution and noninvasive nature [14]. Compared with that of conventional CT, the scan time and radiation dose of CBCT can be significantly reduced [15]. In orthodontics, the replacement of conventional plain radiographs with CBCT appears to be an unavoidable trend, and the cost-benefits analysis demonstrated that CBCT was superior to the combination of several 2D radiographic images with respect to the intrinsic information, the radiation dose and cost [15, 16]. The purpose of this study is to investigate the anatomic features of three-rooted DMSMs in Chinese children by using CBCT. The prevalence rate, distribution pattern, odontometric parameters were examined and compared with those of the three-rooted PMFMs, and the null hypothesis tested was that there was no difference between the three-rooted DMSMs and three-rooted PMFMs.

## Materials and methods

### Subjects

The study protocol has been reviewed and approved by the Ethics Committee of Ninth People's Hospital of Suzhou (Issuing Number: #2017-30). Dental CBCT data (DICOM format), which has been previously obtained in the Dep. of Stomatology, Ninth People's Hospital of Suzhou, China, from June 2017 to December 2021, were retrospectively screen and examined by an endodontist with three years’ experience (*Chengfeng Jiang*). All the examinations were performed for diagnostic reasons before complex oral surgeries or orthodontic treatment. To study the three-rooted DMSMs, the including criteria for subject selection were the following: (1) the age was less than 12 years old; (2) gender known; (3) each subject at least had an unilateral DMSM; (4) teeth with sound dental structures, which allowed for accurate detection of the root number or odontometric measurement of the DB and DL root (otherwise the tooth was recorded as “missing data”). To compare the geometric data of three-rooted DMSMs with those of the three-rooted PMFMs, CBCT scans of 100 adult Chinese subjects were randomly selected from the data base. The selection criteria were the following: (1) The age was greater than 18 years old, and the root apexes had fully matured; (2) gender known; (3) each subject had bilateral PMFMs; (4) the PMFMs had sound dental structures for odotometric measurement. The PMFMs in the children group were excluded from odontometric measurement due to unmatured root apexes and undifferentiated root canal system. The excluding criteria were: (1) The quality of the CBCT images did not allow for accurate detection of the root number or odontometric measurements; (2) teeth with root canal fillings, posts, crowns, coronal or root resorption, extensive coronal and root caries, and periapical/periradicular radiolucency (all recorded as “missing data”). Overall, a total of 247 CBCT scans of children subjects were selected based on inclusion and exclusion criteria.

### CBCT scanning

Imaging was performed using an 3D eXam i (KaVo Co., Germany) CBCT machine. The imaging protocol for adults was as follows: field of view (FOV) = 14 × 8.5 cm; tube peak potential = 120 kVp; tube current = 5 mA; time = 23 s; voxel size = 0.20 mm. For children, the protocol was: FOV = 8.5 × 8.5 cm; tube peak potential = 120 kVp; tube current = 5 mA; time = 23 s; voxel size = 0.20 mm. Thyroid shielding was always used for the pediatric patients.

### Observation and geometric measurement of the DL roots

**Fig. 1:**
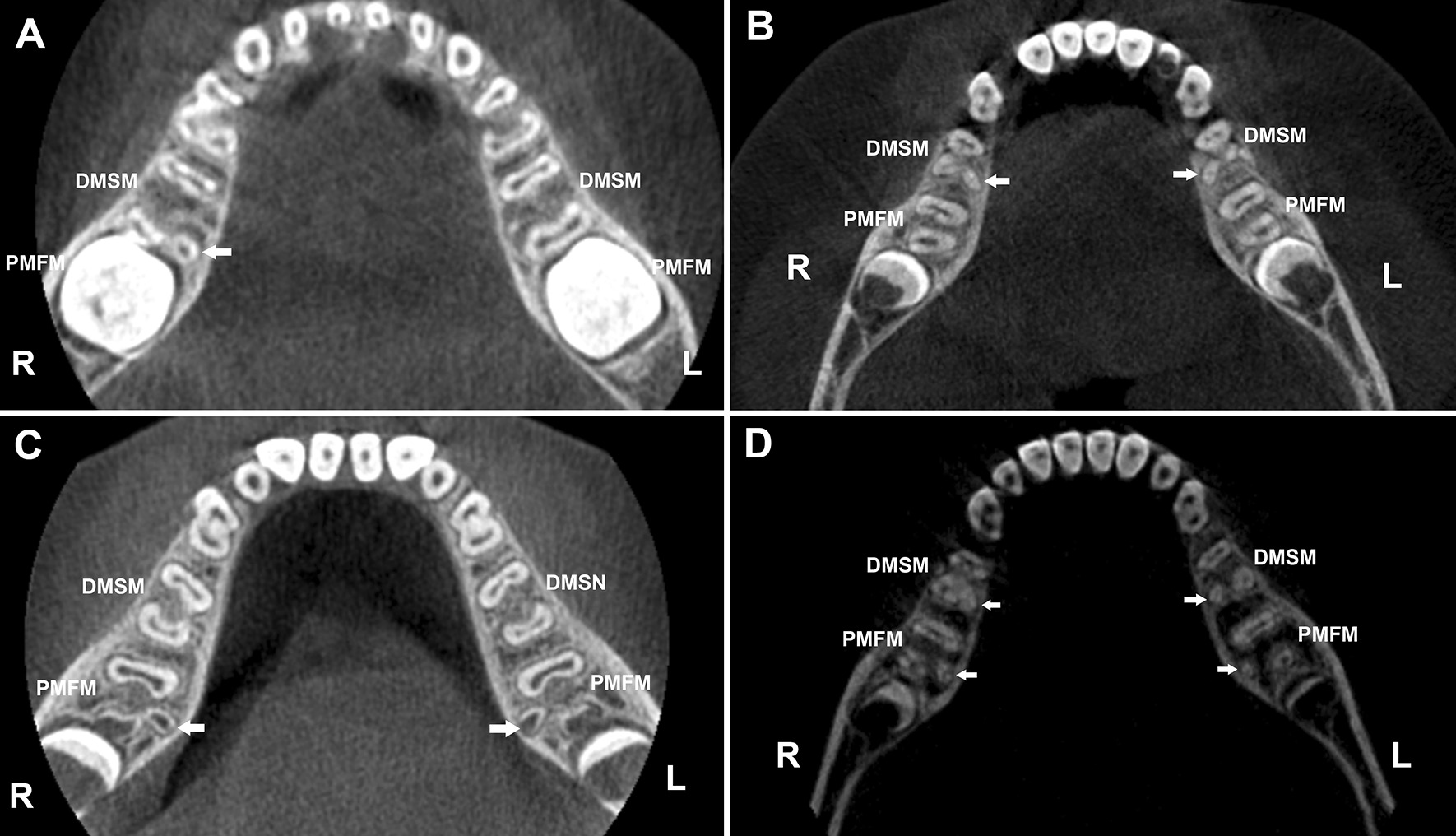
Representative CBCT images of three-rooted mandibular molars (horizontal view). **a** A boy aged six years exhibits a three-rooted DMSM at the right side. **b** A boy aged nine years exhibits bilateral three-rooted DMSMs. **c** A boy aged seven years exhibits bilateral three-rooted PMFMs. **d** A girl aged nine years exhibits bilateral three-rooted DMSMs and bilateral three-rooted PMFMs. DMSM is deciduous mandibular second molar and PMFM is permanent mandibular first molar. Arrows indicate the disto-lingual roots

Kavo eXam Vision software (KAVO, Germany) was used to read the images. By switching the viewing angle (horizontal plane (Fig. 1), mesiodistal plane, buccal-lingual plane, and any other arbitrary planes) and adjusting the magnification, the root anatomy of the DMSMs and PMFMs were examined in detail, and the number of the roots were recorded.

**Fig. 2:**
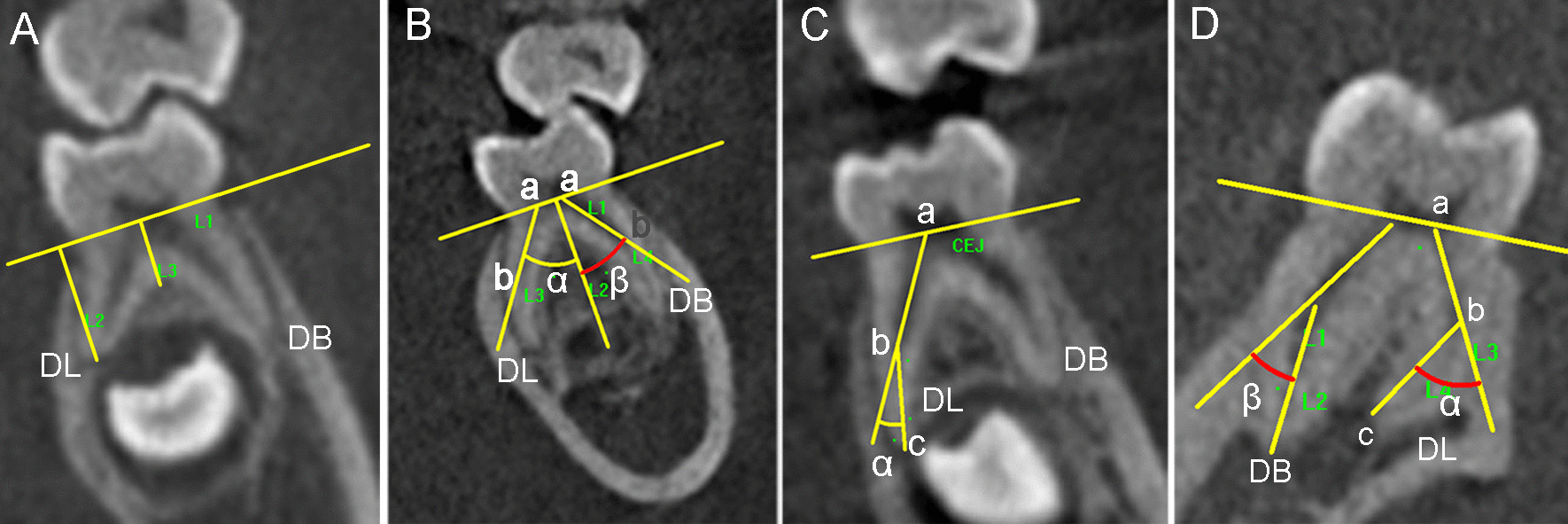
Odontometric measurement of the DB and DL roots of three-rooted deciduous mandibular second molars (DMSMs) and a three-rooted permanent mandibular first molar (PMFM) in the CBCT images (from the adjusted proximal view). **a** L2 is the vertical length of the DL root; L3 is the level of the distal root furcation (a DMSM). **b** Angle α is the spreading angle of DL root, and angle β is the spreading angle of DB root (a DMSM). **c** Angle α is the curvature angle of the DL root measured by Schneider’s method (a DMSM). **d** Measurement of the angle of root curvature of the DL and DB root by Schneider’s method (a PMFM)

To obtain the section of the distal root furcation (between the DB and DL roots) and plane of root curvature (in many cases, the three planes did not coincide), the direction of the distal roots was first adjusted to the vertical position in the clinical view. Then drew an appropriate section line in the horizontal plane to create a new sectional image, which could best display the distal root furcation or the root curvature from the canal orifice to the apex. Saved the sectional image in BMP format, and then software Image-Pro Plus 6.0 (Media Cybernetics, Silver Spring, MD) was used to perform odontometric analysis. After calibration, the vertical length of the root (Fig. 2a), level and angle of the distal root furcation, the spreading angle of DB and DL roots were measured (Fig. 2a, b). The canal curvatures were measured by using Schneider’s method [17]. Briefly, point a was marked at the center of the canal orifice. A line was drawn with a straight edge aligned parallel to axis from point a to a point where the long axis deviated from the straight edge, point b. A third point (point c) was made at the root apex, and a line was drawn from this point to point b. The acute angle formed by the intersection of the two lines was measured to evaluate the canal curvature (Fig. 2c, d). The canal curvatures were separated into three groups on the basis of the angles: straight (10 degrees or less), moderate (10–20 degrees), and severe (20 degrees or more). The spreading angle of DB or DL root was the angle between the line ab (L3 or L4 in Fig. 2b) and the tooth axis (L2 in Fig. 2b), and the angle of distal root furcation was the intersection angle between the L3 and L4 in Fig. 2b, and the value was equal to the sum of the spreading angle of the DB and DL root.

### Statistical analysis

All morphologic assessment and measurements were performed by one examiner (*Chengfeng Jiang*). Intra-observer agreement was estimated using 20 random CBCT images of DL roots (10 DMSMs and 10 PMFMs), and the root curvature was measured twice with an interval of two weeks. Intraclass correlation coefficients (ICC) based on a one-way random effects model were calculated. The ICC for intra-observer agreement was 0.986 (95% CI 0.968, 0.995) (*p* = 0.000), suggesting the measured data were reliable and the error could be ignored.

All odontometric measurement results were reported as means ± SD. The means were compared by Student-*t* test or paired-*t* test. Chi-square test was used to compare the occurrence rates. Spearman’s rank correlation test was used to analyze the relationship between the antimetric three-rooted molars, as well as the relationship between the three-rooted DMSM and three-rooted PMFM at each side. *p* < 0.05 was considered as statistically significant. The SPSS 17.0 software (SPSS, Chicago, IL) was used for statistical analyses.

## Results

### The occurrence rate of the DL roots

The analysis of the total 247 selected scans of children subjects showed that 153 scans (61.9%) were from males and 94 (38.1%) from females, with the age ranging from 5 to11 years (average age = 7.3 ± 1.3 years). Among 247 subjects, 22 subjects have a DMSM unilaterally, and the other 225 subjects have bilateral DMSMs. The occurrence of DL root was 24.0% (54/225) calculated by individual, and 18.6% (88/472) by teeth.

Among 247 children, 231 subjects had bilateral PMFMs. The occurrence of DL root was 29.0% (67/231) calculated by individual and 24.0% (111/462) by teeth. The discrepancy between the DMSMs and PMFMs was statistically significant as accounted by teeth (*p* < 0.05), but not by individuals (*p* > 0.05) (Table 1).

**Table 1:** The occurrence of DL Roots in DMSMs and PMFMs. % (*n/N*)

	DMSMs	PMFMs	*X*^*2*^	*p* value
Male (counted by individual)	24.6% (35/142)	29.2% (42/144)	0.742	0.233
Female (counted by individual)	22.9% (19/83)	28.7% (25/87)	0.756	0.244
Both genders (counted by individual)	24.0% (54/225)	29.0% (67/231)	1.464	0.244
Male (counted by tooth)	19.0% (56/295)	24.3% (70/288)	2.437	0.072
Female (counted by tooth)	18.1% (32/177)	23.6% (41/174)	1.602	0.128
Both genders (counted by tooth)	18.6% (88/472)	24.0% (111/462)	4.033	0.046

*DMSM* deciduous mandibular second molar; *PMFM* permanent mandibular first molar

### Gender difference

In females, the occurrence rate of three-rooted DMSCs was 22.9% (19/83) as calculated by individual and 18.1% (32/177) by tooth. In males, the frequency was 24.6% (35/142 individuals) and 19.0% (56/295 teeth), respectively. Gender difference of DL roots was neither detected in the DMSMs, nor in the PMFMs by both counting methods (all *p* > 0.05) (Table 1).

### Side difference and bisymmetry

The frequency of three-rooted DMSCs was 23.0% (55/239) for the right and 14.2% (33/233) for the left side, and a right-side predilection was detected (*X*^2^ = 6.091, *p* = 0.018). The proportion of bilateral three-rooted DMSMs (Fig. 1b, d) among the three-rooted subjects was 45.7% (16/35) for the males, 55.0% (11/20) for the females, and 49.1% (27/55) for both genders. Spearman’s rank correlation test shows there is a significant co-relationship between the antimetric teeth (*rho* = 0.609, *p* < 0.01) (Table 2).

**Table 2:** Bisymmetry of the three-rooted deciduous mandibular second molars (*n*)

	Male			Female			Both genders		
	L^P^	L^A^	total	L^P^	L^A^	total	L^P^	L^A^	Total
R^P^	16	17	33	11	6	17	27	23	50
R^A^	2	107	109	3	63	66	5	170	175
total	18	124	142	14	69	83	32	193	225
*Spearman’s rank correlation test*									
	*rho* = 0.607, *p* = .000			*rho* = 0.648, *p* = .000			*rho* = 0.609, *p* = .000		

R: right; L: left; ^P^: presence of the DL root; ^A^: absence of the DL root

The frequency of three-rooted PMFMs (Fig. 1c, d) was 26.8% (62/231) for the right and 21.2% (49/231) for the left side, and the side difference has no statistical significance (*X*^2^ = 2.004, *p* = 0.191).

### The relationship of occurrence between the three-rooted DMSMs and three-rooted PMFMs

The concurrence rate of three-rooted DMSMs and adjacent three-rooted PMFMs (Fig. 1d) was 31.6% (24/76) for the right and 23.0% (14/61) for left side. Spearman’s rank correlation test shows there is a weak but statistically significant relationship between the two teeth (right side: *rho* = 0.325, *p* < 0.01; left side: *rho* = 0.260, *p* < 0.01) (Table 3).

**Table 3:** Concurrence of the three-rooted DMSMs and PMFMs (*n*)

	Right			Left		
	DMSM^P^	DMSM ^A^	Total	DMSM ^P^	DMSM^A^	Total
PMFM^P^	24	33	57	14	32	46
PMFM^A^	19	132	151	15	156	171
Total	43	165	208	29	188	217
*Spearman’s rank correlation test*						
	*rho* = 0.325, *p* = .000			*rho* = 0.260, *p* = .000		

DMSM: deciduous mandibular second molar; PMFM: permanent mandibular first molar; ^P^: presence of the DL root; ^A^: absence of the DL root

### The odontometric analysis of the DL and DB roots

**Fig. 3:**
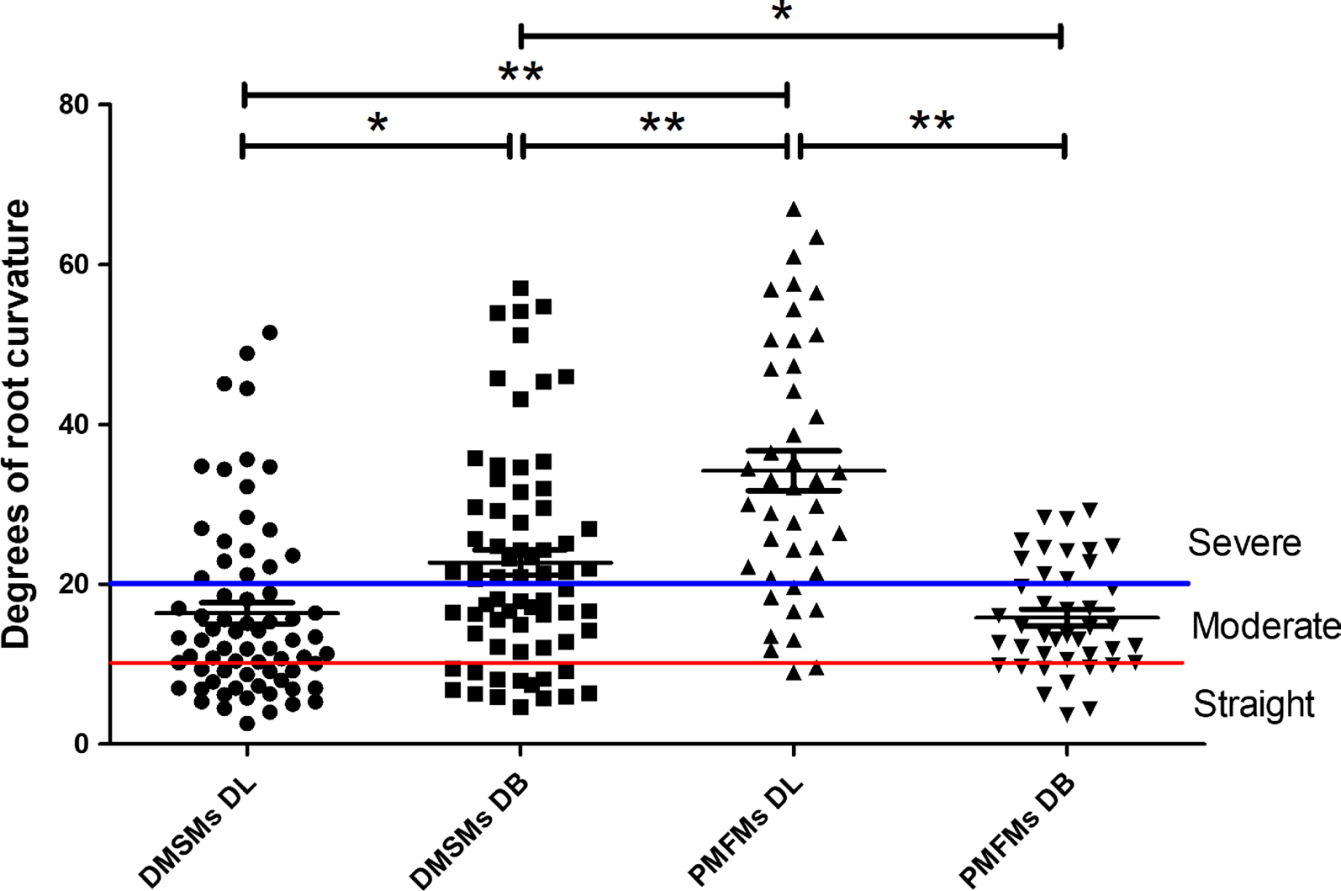
Degrees of root curvatures in the disto-lingual (DL) and disto-buccal (DB) roots of the three-rooted deciduous mandibular second molars (DMSMs) and three-rooted permanent mandibular first molars (PMFMs). Measured by Schneider’s technique. Error bar is SEM, *p < 0.05, **p < 0.01

The odontometric data of three-rooted DMSMs and three-rooted PMFMs were shown in Table 4 and Fig. 3. Among total 88 three-rooted DMSMs detected in the children group, odontometric analysis was performed on 70 teeth and the other 18 molars were excluded from measurement due to root absorption. Among 100 adult subjects with bilateral PMFMs, the DL roots were detected in 27 individuals and 42 PMFMs. For the three-rooted DMSMs, the mean values of the DL and DB root length, the level of the distal root furcation, as well as the degrees of the DL root curvature were significantly less than those of the three-rooted PMFMs (all *p* < 0.01). Whereas, the DMSMs displayed greater root spreading angle, furcation angle and DB root curvature as compared to the PMFMs (all *p* < 0.01). In DMSMs, the DL roots were less curved than the DB roots (mean degrees of curvature: 16.4 degrees vs 22.7 degrees; proportion of severe curvature: 27.1% [19/70] vs 51.4% [36/70]; both *p* < 0.01); while in PMSMs, the DL roots were significantly more curved than the DB roots (mean degrees of curvature: 34.2 degrees vs. 15.8 degrees; proportion of severe curvature: 78.6% [33/42] vs 28.6% [12/42], both *p* < 0.01).

**Table 4:** Comparison of the odontometric data of the three-rooted DMSMs and three-rooted PMFMs

	DMSMs (*n* = 70) $\overline{X}$ ± *S*	PMFMs (*n* = 42) $\overline{X}$ ± *S*	*t* value	*p* value
Length of DB root (mm)	8.4 ± 1.5	12.3 ± 1.3	14.27	< 0.001
Length of DL root (mm)	7.4 ± 1.5	11.0 ± 1.3	13.06	< 0.00
Level of distal root furcation (mm)	3.3 ± 0.8	4.6 ± 0.8	8.45	< 0.001
Spreading angle of DB root (degrees)	32.8 ± 8.7	19.9 ± 5.9	8.53	< 0.001
Spreading angle of DL root (degrees)	34.6 ± 8.4	26.8 ± 6.5	5.18	< 0.001
Angle of distal root furcation (degrees)	67.4 ± 14.4	46.7 ± 9.5	8.30	< 0.001
Angle of curvature of DB root (degrees)	22.7 ± 13.5	15.8 ± 6.8	3.08	< 0.01
Angle of curvature of DL root (degrees)	16.4 ± 11.3	34.2 ± 16.1	6.89	< 0.001

*DMSM* deciduous mandibular second molar, *PMFM* permanent mandibular first molar

In view of the significant difference in the occurrence rate (counted by teeth) and odontometric data between the three-rooted DMSMs and PMFMs, the null hypothesis tested was rejected.

## Discussion

This in vivo study investigated the anatomic features of three-rooted DMSMs by using CBCT. Although the sample size is smaller than several previous studies based on panoramic [13] or periapical radiographs [18], CBCT allows for more accurate detection of the DL root and non-invasive 3D odontometric analysis, especially can provide valuable anatomic information from the proximal view.

We demonstrated that the occurrence of three-rooted DMSMs is high in the Chinese children, and 24.0% of the included subjects, and 18.6% of examined teeth exhibited the DL root. The percentages are slightly lower than those reported by Hsu et al. [13] in a Taiwanese population and Song et al. [19] in a Korea population. Hsu et al. [13] reported that the overall prevalence of three-rooted DMSMs was 28.4% (168/591) as calculated by subjects, and 22.4% (265/1182) by teeth, whereas Song et al. [19] reported a very similar percentage of 27.8% (by teeth) based on periapical radiographs. However, the percentage in this work is significantly greater than 10% in another Taiwanese population [20], and it is also greater than 5.6–6.5% in the Indian populations [21, 22]. Martin et al. [14] reported that the incidence of three-rooted PMFMs was only 2.6% (12/466) in whites, as compared with a much higher incidence of 25.9% (57/220) in Asians. In our study, the occurrence rate of the three-rooted DMSMs is significantly lower than that of three-rooted PMFMs (18.6% vs. 24.0%, *p* < 0.05), which has not been reported by Hsu et al. [13] in a Taiwan Chinese population. The data are useful for clinicians to predict the presence of a DL root in the Chinese patients.

In current study, gender predilection was neither detected in the DMSMs, nor in the PMFMs (both *p* > 0.05), which is consist with the findings of Liu et al. [20] and Hsu et al. [13] in the Taiwanese populations, but in contrast with Song et al. [19], who reported a significant gender predilection in males over females (*p* < 0.05). Side predilection has been reported previously on three-rooted PMFMs in different populations, and there are more reports of a right-side predilection [7, 11, 18, 19, 23, 24] than those of a left-side predilection [4, 25]. We detected a significant right-side predilection in the three-rooted DMSMs (23.0% vs 14.2%, *p* < 0.05), but not in the three-rooted PMFMs.

The bilateral concurrence rate of three-rooted DMSMs is 49.1% (27/55), and the percentage is lower than previous reports on Asian populations (53.7–68.6%) [7, 11, 18, 19, 26]. A significant co-relationship was detected between two sides (*rho* = 0.609, *p* < 0.01). The data can used to predict the presence of an DL root in the antimetric tooth. Following this idea, we calculated the relationship of the occurrence between three-rooted DMSMs and three-rooted PMFMs. We found the concurrence rate was only 31.6% at the right and 23.0% at the left side, and a weak but significant relationship (right side: *rho* = 0.325, *p* < 0.01; left side: *rho* = 0.260, *p* < 0.01) was detected between the two molars. Therefore, we could not confidently predict the presence/absence of the DL root in a PMFM according to the root number of its adjacent DMSM, and vice versa. This finding contradicts that of Hsu et al. [13] who reported that the presence of three-rooted DMSMs can strongly predict the possibility of three roots in PMFMs. The discrepancy can be due to the different geological regions and methodology. The presence of three-rooted DMSMs may pose challenges in pediatric endodontics. Failure to detect, or unproper management of the DL root canal may lead to treatment failure. CBCT scanning was not a routine examination for root canal therapy of deciduous teeth, while the conventional periapical radiography has limitations in the diagnosis of the DL root due to image superimposition of the two distal roots. Therefore, clinicians should aware of the prevalence rate and distribution pattern of this root variation in different populations during dental treatment.

Previous scholars had reported that the DL root of the PMFMs was typically conical and small, and the apex frequently swings towards buccal, and this may pose an endodontic challenge [2, 10, 27]. The current study shows the DL roots in the DMSMs are shorter and less curved as compared to those of the PMFMs, which may be in favor of endodontic treatment. However, because the root trunk is shorter (corresponding to a higher furcation level) and the spreading angle of the distal roots is greater, overzealous drilling or instrumentation may easily lead to perforation at the pulp floor or root furcation area, and further cause damage to the underlying permanent tooth buds. Moreover, the thickness of the canal wall is very small (Fig. 1), therefore, protection of the remaining dental tissue is always the priority during endodontic treatment of the three-rooted DMSMs. With regard to root canal instrumentation, the traditional K-files were usually associated with a prolonged procedure or procedural errors (such as ledging, zipping and canal transportation). In recent years, a great number of novel NiTi rotary systems have been introduced to the market and applied on deciduous molars, which can significantly reduce preparation time, thereby increasing children’s cooperation during treatment [28]. Moreover, their enhanced flexibility can decrease procedural errors and other complications. The single-file NiTi systems show more superior advantages in shortening the instrumentation time and reduce cross-contamination risk. Pawar et al. [28] reported that, XP-endo Shaper (FKG Dentaire, La Chaux-deFonds, Switzerland), an adaptive single-file system, was a favorable technique for pulpectomy of primary molars considering the significant reduction in instrumentation time and better obturation. The file was specifically designed to adapt to the oval or robbin-shaped canal, which seems more suitable for the mesial root canal system of DMSMs. Whereas other non-adaptive single files, often have a fixed shape and taper, prone to create a space representing their shape, and they are expected to be more effective in narrow canals with a circular cross-section [28]. Theoretically, they have advantages in preparing the tiny round DL canals. However, further clinical investigations are essential to verify our speculation.

This study has several limitations. First, the sample size is still relatively small because CBCT examinations should be carried out prudently on children and follow the ALARA principle (as low as reasonably achievable). Reducing the height of the field of view and shielding the thyroid are advisable methods and must be implemented to lower the exposure dose [16]. Second, many subjects were examined by CBCT for orthodontic reasons, which may lead to a biased sample. Third, limited by the functions of the software, the geometric measurement of the DL and DB roots was performed from an adjusted proximal view, which is not a real “3D” measurement. Further studies are required basing on lager sample size and more populations of other ethnic/geological background.

## Conclusions

In conclusion, three-rooted DMSMs display a high occurrence rate in the Chinese people with a right-side predilection, and they have a weak but statistically significant correlation with three-rooted PMFMs. The DL roots of DMSMs are shorter, less curved, and spreading more widely as compared to those of PMFMs. A better understanding of their anatomic features is essential for successful pediatric endodontic treatment.

## Data Availability

All the datasets used and analyzed during the current study are available from the corresponding author on reasonable request.

## Abbreviations

CBCT: Cone-beam computed tomography
PMFM: Permanent mandibular first molar
DMSM: Deciduous mandibular second molar
DB: Disto-buccal
DL: Disto-lingual
3D: Three-dimensional
